# The effect of noninvasive brain stimulation on anhedonia in patients with schizophrenia and depression: A systematic review and meta‐analysis

**DOI:** 10.1002/pchj.723

**Published:** 2023-12-27

**Authors:** Min‐yi Chu, Shuai‐biao Li, Yi Wang, Simon S. Y. Lui, Raymond C. K. Chan

**Affiliations:** ^1^ Shanghai Mental Health Center Shanghai Jiao Tong University School of Medicine Shanghai China; ^2^ Neuropsychology and Applied Cognitive Neuroscience Laboratory CAS Key Laboratory of Mental Health, Institute of Psychology, Chinese Academy of Sciences Beijing China; ^3^ Department of Psychology University of Chinese Academy of Sciences Beijing China; ^4^ Department of Psychiatry, School of Clinical Medicine The University of Hong Kong Hong Kong China

**Keywords:** anhedonia, depression, meta‐analysis, noninvasive brain stimulation, schizophrenia

## Abstract

Anhedonia is a transdiagnostic symptom found in patients with schizophrenia and depression. Current pharmacological interventions for anhedonia are unsatisfactory in a considerable proportion of patients. There has been growing interest in applying noninvasive brain stimulation (NIBS) to patients with anhedonia. However, evidence for the efficacy of NIBS for anhedonia remain inconsistent. This study systematically identified all studies that measured anhedonia and applied NIBS in patients with schizophrenia or depression. We conducted a search using the various databases in English (PubMed, EBSCOHost (PsycInfo/PsycArticles), Web of Science) and Chinese (China National Knowledge Infrastructure, Wanfang Data Knowledge Service Platform) languages, and reviewed original research articles on NIBS published from January 1989 to July 2023. Our search had identified 15 articles for quantitative synthesis, with three concerning schizophrenia samples, 11 concerning samples with depression, and one concerning both clinical samples. We conducted a meta‐analysis based on the 15 included studies, and the results suggested that NIBS could improve anhedonia symptoms in schizophrenia patients and patients with depression, with a medium‐to‐large effect size. Our findings are preliminary, given the limited number of included studies. Future NIBS research should measure anhedonia as a primary outcome and should recruit transdiagnostic samples.

## INTRODUCTION

Anhedonia is a transdiagnostic symptom for many psychiatric disorders, particularly schizophrenia and depression (Lambert et al., 2018; Liang et al., 2022). According to *DSM‐5*, anhedonia is a core symptom of schizophrenia and depression. Moreover, this symptom strongly predicts poor clinical and functional outcomes in patients with depression and schizophrenia (Spano et al., 2019). There has been growing interest in developing effective interventions for anhedonia. For schizophrenia patients, atypical antipsychotics appear to be more effective for anhedonia than typical antipsychotics, but a considerable proportion of schizophrenia patients exhibit disabling anhedonia symptoms after medication treatment (Liang et al., 2022). For patients with depression, anhedonia can also be resistant to many first‐line antidepressants (Fusar‐Poli et al., 2015).

Classically, anhedonia refers to the reduced ability to experience pleasure. Recent research conceptualized anhedonia as a multidimensional construct, comprising at least two components, i.e., anticipatory anhedonia (which refers to the diminished motivation to pursue rewards) and consummatory anhedonia (which refers to the diminished hedonic response to rewards) (Treadway & Zald, 2011; Zhang et al., 2016). The transdiagnostic approach further investigated the commonalities and differences in anhedonia between schizophrenia patients and patients with depression. Although both schizophrenia patients and patients with depression showed anticipatory and consummatory anhedonia, such symptoms apparently constituted a “state” in patients with depression (i.e., anhedonia may fluctuate with the severity of depressive symptoms, illness duration, and relapses); but anticipatory anhedonia appeared to be a “trait” in schizophrenia patients (Li et al., 2015). Interestingly, some studies suggested that schizophrenia and depression may share similar neurobiological alterations in the frontostriatal and mesocorticolimbic circuits underlying the reward system (Lambert et al., 2018; Liang et al., 2022; Zhang et al., 2016). Such shared neural dysfunctions may explain anhedonia symptoms found in the two different disorders, and the specific brain circuits involved may be targets for neuromodulations.

Noninvasive brain stimulation (NIBS) is a non‐pharmacological technique for treating anhedonia. Research suggests that NIBS shows promise for reducing anhedonia in various neurological and psychiatric disorders (Camacho‐Conde et al., 2022; Homan et al., 2021; Tseng et al., 2022). Transcranial magnetic stimulation (TMS) may involve the application of a single pulse, paired pulses, or repetitive pulses to patients, and has been used in research and clinical settings (Bhattacharya et al., 2022). Repetitive TMS (rTMS) has been widely used in various mental disorders, including schizophrenia, depression, and anxiety disorders (Rossi et al., 2009; Shi et al., 2014). For instance, rTMS to the targeted brain cortical regions could induce a magnetic field, which would create rapidly alternating electric current a few centimeters underneath the scalp. It could bring changes in the amplitudes of motor evoked potentials (Jannati et al., 2023; Marder et al., 2022). In general, high‐frequency (≥5 Hz) rTMS and intermittent theta‐burst stimulation (iTBS) would enhance cortical excitability, whereas low frequency (1 Hz) rTMS and continuous theta‐burst stimulation (cTBS) would inhibit cortical excitability (Bhattacharya et al., 2022). Transcranial direct current stimulation (tDCS) and transcranial alternating current stimulation (tACS) are different type of NIBS (Li et al., 2022; Muszkat et al., 2016). The tDCS technique applies a weak (1–2 mA) direct current across the scalp to enhance (anode) or inhibit (cathode) cortical excitability (Nitsche et al., 2008). Moreover, compared to traditional tDCS with large electrode pads, high‐definition tDCS (HD‐tDCS) could increase the focality of stimulation and could prolong the sustainability of its effects (Alam et al., 2016; Kuo et al., 2013; To et al., 2016). On the other hand, tACS is a relatively novel technique, which applies a sinusoidal oscillating current to modulate the ongoing rhythmic activity of the brain (Fiene et al., 2020; Zaehle et al., 2010). Although the mechanisms of the aforementioned NIBS techniques are different, all could enhance brain plasticity (Bhattacharya et al., 2022).

There has been growing interest in applying NIBS to alleviate anhedonia in schizophrenia patients and patients with depression (Liang et al., 2022; Spano et al., 2019). A recent systematic review by Spano and colleagues explored the potential impact of neuromodulation interventions on anhedonic traits in clinical and nonclinical samples (Spano et al., 2019). Only one study on schizophrenia patients and two studies on patients with depression were included in Spano et al.'s review. Moreover, all these three included studies used rTMS. More recent rTMS studies on anhedonia in schizophrenia and depression samples showed mixed results. For instance, several recent studies on schizophrenia patients reported that rTMS effectively improved anhedonia symptoms (Gan et al., 2021; Kumar et al., 2020; Prikryl et al., 2013), but one study reported a non‐significant effect (Bodén et al., 2021). For patients with depression, some studies reported significant effects of rTMS on anhedonia (Bodén et al., 2021; Wang et al., 2021), but others did not find any improvement after rTMS (Diederichs et al., 2021; Duprat et al., 2018). The effects of tDCS on anhedonia also remains unclear. For instance, Jog and colleagues found the anodal HD‐tDCS significantly improved anhedonia symptoms in patients with depression, but no significant improvement was found in those patients who received conventional tDCS (Jog et al., 2021). However, another recent study found that conventional tDCS could effectively improve anhedonia in depression patients (Zhu et al., 2022).

Taken together, the evidence remains inconclusive regarding the potential therapeutic benefit of NIBS on anhedonia in schizophrenia patients and patients with depression. In view of the adverse effect of anhedonia symptoms in the outcome of schizophrenia and depression, and the current limitations of pharmacological interventions on anhedonia, it is necessary to review the current evidence for NIBS on anhedonia in schizophrenia and depression. In this systematic review and meta‐analysis, we searched comprehensively the previous studies that applied NIBS to patients with schizophrenia or depression and measured anhedonia as an outcome. We aimed to conduct qualitative and quantitative analyses to ascertain the NIBS effect on anhedonia in these two clinical populations. We hypothesized that the NIBS would improve anhedonia in schizophrenia and depression.

## METHODS

### Literature search

We searched four databases in the English language (i.e., PubMed, EBSCOHost (PsycInfo/PsycArticles), Web of Science) and two in the Chinese language (i.e., China National Knowledge Infrastructure, Wanfang Data Knowledge Service Platform) for relevant articles published from January 1989 to July 2023. The English keywords were “anhedonia”, “pleasure”, “SHAPS”, “hedonic”, “neuromodulation”, “noninvasive brain stimulation”, “transcranial magnetic stimulation”, “repetitive transcranial magnetic stimulation”, “direct transcranial current stimulation”, “transcranial alternating current stimulation”, “tDCS”, “TMS”, “rTMS”, “tACS”. The Chinese key words were “快感缺失”, “愉悦情绪”, “SHAPS”, “愉快感”, “神经调控”, “非侵入性脑刺激”, “经颅磁刺激”, “重复经颅磁刺激”, “经颅直流电刺激”, “经颅交流电刺激”, “tDCS”, “TMS”, “rTMS”, “tACS”. The search strategy is detailed in Data S1. In addition, we conducted a manual search by reviewing the reference lists of relevant articles. Finally, our search strategy yielded 375 potential articles for inclusion.

### Study selection

**FIGURE 1:**
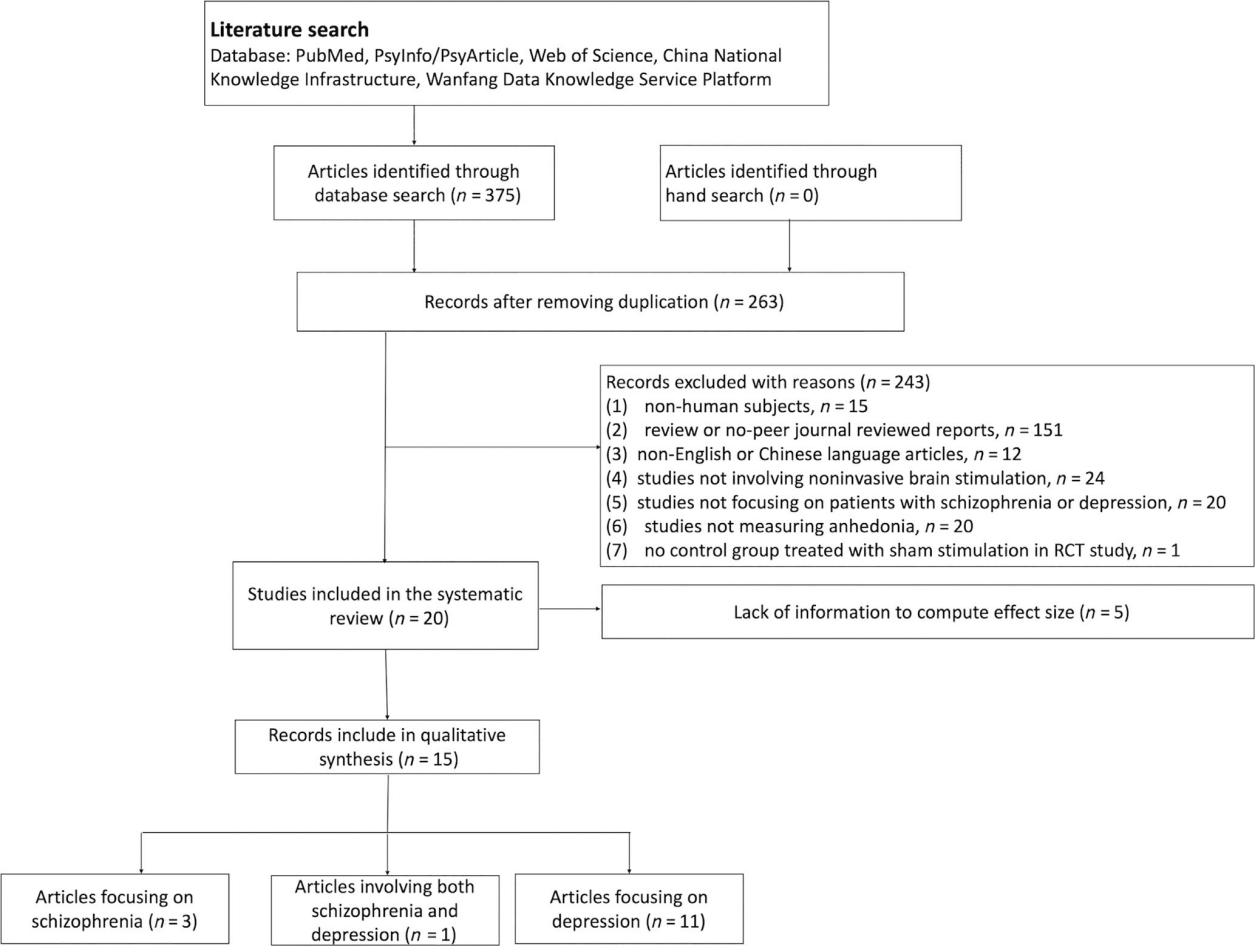
Flow diagram of article selection.

Articles were eligible according to the following inclusion criteria: (1) study type: randomized controlled trial (RCT) or single‐arm trail; (2) participants: patients having a diagnosis of schizophrenia or MDD; (3) intervention: an experimental group treated with NIBS (tDCS, rTMS, or tACS), and a control group treated with sham stimulation (in RCT studies); and (4) outcome measures: a valid measure of anhedonia commonly used in schizophrenia or MDD, including the anhedonia subscale of Assessment of Negative Symptoms (SANS), the Clinical Assessment Interview for Negative Symptoms (CAINS), the Snaith‐Hamilton Pleasure Scale (SHAPS), the Dimensional Anhedonia Rating Scale (DARS), the Temporal Experience of Pleasure Scale (TEPS), the Chapman physical and social anhedonia questionnaire. The reasons for exclusion of irrelevant studies are presented in Figure 1.

### Data extraction

For each study, we recorded the following variables: (1) publication information (the name of the first author and years of publication); (2) sample size; (3) study design; (4) neuromodulation interventions and protocols (type, target area, number of sessions); and (5) instruments for measuring anhedonia. To calculate the effect sizes for the meta‐analysis, we extracted the means, SDs, and samples size of the pre‐ and post‐treatment of one group (active group in the open‐label study) or two independent groups (active and sham group in the sham‐controlled study). When the means, SD, and sample size were unavailable, the effect size or mean changes in pre‐ to post‐treatment of the group were extracted for data encoding. If the articles did not report sufficient data and information for estimating the effect size, we would contact the authors via email. All information was extracted by one author (CMY) and independently confirmed by another author (LSB).

### Quality assessment

Two authors (CMY and LSB) independently assessed the methodological quality of the RCT studies using the Cochrane Collaboration software, Review Manager (RevMan) 5.3 (Higgins & Altman, 2008), with seven domains: random sequence generation, allocation concealment, blinding of participants and personnel, incomplete outcome data, selective reporting, and other bias. Each bias domain was judged as low, unclear, or high. Certainty of evidence and summary of findings were also independently assessed by two reviewers (CMY and LSB) according to the recommendation of the *Cochrane Handbook* (Hultcrantz et al., 2017). Five criteria were used to judge the certainty of evidence: risk of bias, inconsistency, indirectness, imprecision, and publication bias.

### Data analysis

All analyses were performed using the Comprehensive Meta‐analysis (CMA) software package (Version 3). The effect size was calculated as Hedges' *g*, indexing the difference of pre‐ and post‐treatment values of one group (active group) or comparison of the mean changes in pre‐ to post‐treatment scores between active and sham groups. The random effect model was used to compute the effect size (Hedges' *g*). *Q*‐test and *I* ^2^ were performed to assess the heterogeneity of the studies included in our pooled estimates. For one study that provided two indexes on anhedonia, we first calculated the effect size for each index and then combined them as a single mean weighted effect size (Zhu et al., 2022). Another study used two types of stimulated intervention (high definition or conventional tDCS) with different samples (Jog et al., 2021). We separately calculated the effect size of the two interventions as independent studies. In one included study (Duprat et al., 2018), a cross‐over design was used, so that participants received either active or sham stimulation in the first week but then crossed over to the opposite treatment arms in the second week (Duprat et al., 2018). To control for the potential bias of a cross‐over design, we only calculated the severity of anhedonia before and after the first week of stimulation for active and sham conditions in that included study.

## RESULTS

A total of 375 potential papers were obtained from Pubmed, PsyInfo/PsyArticle, Web of Science, China National Knowledge Infrastructure, and Wanfang Data Knowledge Service Platform. We also manually screened the reference lists of several relevant review articles (Gupta et al., 2018; Li et al., 2022; Siddiqi et al., 2022; Spano et al., 2019). Finally, 20 studies (19 English papers and one Chinese paper) were retained after excluding those articles that failed to meet the eligibility criteria. Given that five articles did not contain sufficient information for the estimation of effect size (Dhami et al., 2019; Downar et al., 2014; Kaboodvand et al., 2023; Light et al., 2019; Rezaei et al., 2021), we only included 15 articles for quantitative synthesis. Among them, three concerned schizophrenia patients (Gan et al., 2021; Kumar et al., 2020; Prikryl et al., 2013), 11 concerned patients with depression (Diederichs et al., 2021; Duprat et al., 2018; Elemery et al., 2022; Fukuda et al., 2021; Jog et al., 2021; Krepel et al., 2020; Lazary et al., 2021; Rezaei et al., 2023; Russo et al., 2018; Wang et al., 2021; Zhu et al., 2022), and one concerned both clinical groups (Bodén et al., 2021). Three studies on patients with depression combined NIBS with medications or psychological interventions (Krepel et al., 2020; Russo et al., 2018; Zhu et al., 2022), and another study recruited patients with bipolar depression (Diederichs et al., 2021). Considering the small number of identified studies, we retained these studies for meta‐analysis. Figure 1 illustrates the search and selection processes.

**FIGURE 2:**
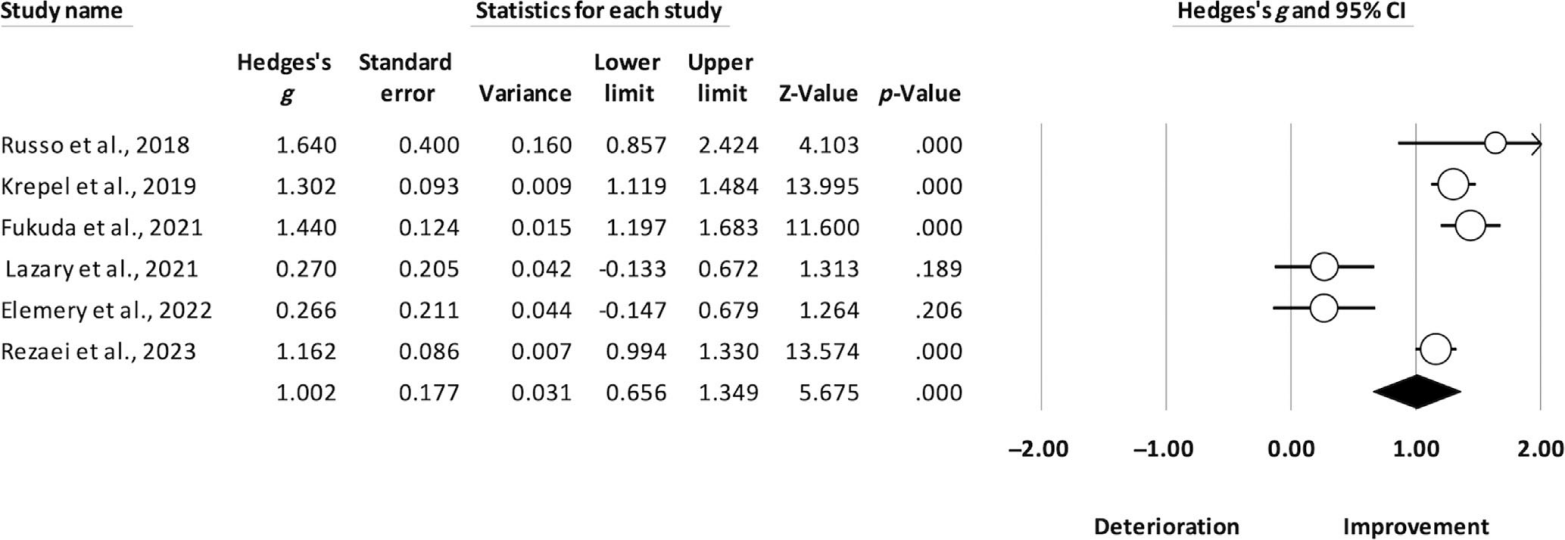
Forest plot for patients with schizophrenia. Heterogeneity: *Q* = 7.820, *df* = 3, *p* = .005, *I* ^2^ = 61.64.

**FIGURE 3:**
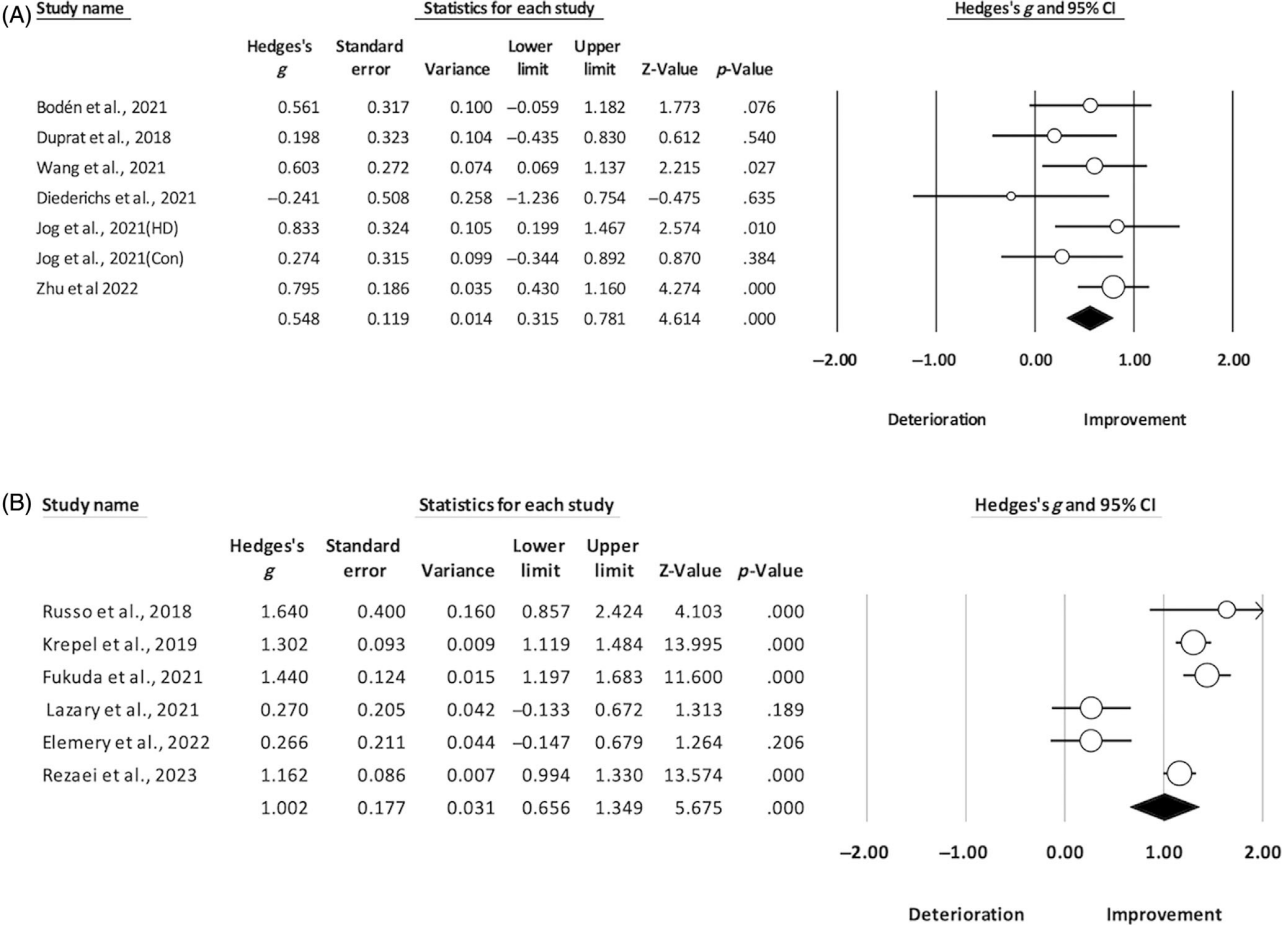
Forest plot for patients with depression. Panel A shows the results of sham‐controlled studies. Heterogeneity: *Q* = 6.902, *df* = 6, *p* = .330, *I* ^2^ = 13.073. Panel B shows the results of open‐label studies. Heterogeneity: *Q* = 45.626, *df* = 5, *p* < .001, *I* ^2^ = 89.041.

The risk of bias is reported in Figures S1 and S2. The GRADE assessments indicated a very low to low level of certainty (see Table S1). Tables 1 and 2 summarize the effect size of each study investigating the therapeutic effect of NIBS on anhedonia in schizophrenia and depression patients. Regarding schizophrenia, all of the four articles were sham‐controlled design. The random effects model showed a mean effect size (Hedges' *g*) of 0.665 (95% confidence interval [CI]: [0.130, 1.201], *p* = .015), indicating that, compared to the sham condition, NIBS appeared to be effective in reducing anhedonia in schizophrenia, with a medium‐to‐large effect size (see Figure 2). Regarding patients with depression, we included 12 articles, with six open‐label and six shamed‐controlled interventions. As shown in Figure 3A, the mean effect size (Hedges' *g*) was 0.548 (95% CI [0.315, 0.781], *p* < .001) when we compared the pre‐post changes between active and sham stimulation based on the six sham‐controlled studies. Similarly, we also analyzed the effects of pre‐post intervention on anhedonia based on the four open‐label studies, and we found a mean effect size (Hedges' *g*) of 1.002 (95% CI [0.656, 1.349], *p* < .001) (see Figure 3B).

**TABLE 1:** Descriptions of studies in schizophrenia patients.

Study				Stimulation parameters						Measure tools	Hedges' *g* (95% CI)
Authors	Design	Sample size (active: sham)		Stimulation intervention	Target area	Frequency	%MT	No. of session	Number of impulses per session		
		Baseline	Post intervention								
Prikryl et al., 2013	Parallel	25:20	23:17	rTMS	Left DLPFC	10 HZ	110% (resting)	15	2000	SANS anhedonia subscale	0.975 (0.324, 1.625)
Kumar et al., 2020	Parallel	50:50	46:47	rTMS	Left DLPFC	20 HZ	100% (NA)	20	2000	SANS anhedonia subscale	0.387 (−0.019, 0.794)^a^
Gan et al., 2021	Parallel	17:16	15:14	rTMS	DMPFC	10 HZ	100–120% (resting)	20	6000	SANS anhedonia subscale	1.351 (0.562, 2.139)
Bodén et al., 2021	Parallel	9:7	NA	iTBS	DMPFC	—	90% (resting)	20	1200	CAINS	−0.077 (−1.012, 0.857)^b^

Abbreviation: DMPF: dorsomedial prefrontal cortex; DLPFC: dorsolateral prefrontal cortex; NA: not available.

^a^ The effect size was computed with the baseline data including the participants who withdrew the intervention.

^b^ The effect size was computed by the sample size of post‐intervention.

**TABLE 2:** Descriptions of studies in depression patients.

Study				Stimulation parameters						Measure tools	Hedges's *g* (95% CI)
Authors	Design	Sample size (active: sham)		Stimulation intervention	Target area	Frequency	%MT	No. of session	Number of impulses per session		
		Baseline	Post intervention								
Duprat et al., 2018	Cross‐over	18:19	18:19	iTBS	Left DLPFC	—	110% (resting)	20	1620	TEPS	0.198 (−0.435, 0.830)
Wang et al., 2021	Parallel	33:27	32:24	rTMS	Left DLPFC	10HZ	100% (resting)	15	3000	TEPS	0.603 (0.069, 1.137)^a^
Jog et al., 2021	Parallel	23:11	20:10	HD‐tDCS	Left DLPFC	2 mA (anode)	—	12	—	SHAPS	0.833 (0.199, 1.467)
		22:10	19:10	Cov‐tDCS							0.274 (−0.344, 0.892)
Bodén et al., 2021	Parallel	19:21	NA	iTBS	DMPFC	—	90% (resting)	20	1200	CAINS	0.561 (−0.059, 1.182)^b^
Diederichs et al., 2021	Parallel	11:10	6:8	iTBS	Left DLPFC	—	120% (resting)	20	600	SHAPS	−0.241 (−1.236, 0.754)^a^
Zhu et al., 2022	Parallel	30:31	30:31	Cov‐tDCS	left DLPFC	2 mA (anode)	—	20	—	SHAPS DARS	0.795 (0.430, 1.160)
Russo et al., 2018	Open‐label	11	11	rTMS	Left DLPFC	10 Hz/5 Hz	120 (NA)	33.0 ± 5.7	3000	SHAPS	1.640 (0.857, 2.424)
Krepel et al., 2020	Open‐label	196	170	rTMS	Bilateral method^c^		110–120% (resting)	20.9 ± 7.5	1200 right side 1500 left side	BDI‐II 2,4,21	1.302 (1.119, 1.484)
Fukuda et al., 2021	Open‐label	144	105	rTMS	Left DLPFC	10 Hz/5 Hz	120% (resting)	35.49 ± 6.65	3000–4000	SHAPS	1.440 (1.197, 1.683)
Lazary et al., 2021	Open‐label	18	18	rTMS	Bilateral method^c^		NA	10	990 right side 2000 left side	SHAPS	0.270 (−0.133, 0.672)
Elemery et al., 2022	Open‐label	17	17	rTMS	Bilateral method^c^		NA	10	990 right side 2000 left side	SHAPS	0.266 (−0.147, 0.679)
Rezaei et al., 2023	Open‐label	182	182	Cov‐tDCS	Left DLPFC	2 mA (anode)	NA	10	—	SHAPS	1.162 (0.994, 1.330)

Abbreviations: DMPF: dorsomedial prefrontal cortex; DLPFC, dorsolateral prefrontal cortex; Cov‐tDCS, Conventional montage tDCS; HD‐tDCS, high‐definition tDCS; NA, not available; rTMS, Repetitive TMS.

^a^ The effect size was computed with the baseline data including the participants who withdrew the intervention.

^b^ The effect size was computed by the sample size received allocated intervention.

^c^ 10 Hz on left DLPFC and 1 HZ on right DLPFC.

## DISCUSSION

Our study systematically explored the potential effect of NIBS on anhedonia in patients with schizophrenia and depression. Our quantitative review indicated that, relative to sham condition, NIBS appeared to be effective in reducing anhedonia in patients with schizophrenia, with a medium‐to‐large effect size. Moreover, in patients with depression, open‐label studies collectively indicated a large effect size for NIBS on anhedonia. Regarding the placebo effect, evidence suggested a medium effect of sham‐controlled studies.

Anhedonia can be enduring and difficult‐to‐treat, but no targeted treatment for anhedonia has been approved by the Food and Drug Administration (FDA) (Lambert et al., 2018). We addressed the paucity of evidence by conducting this first meta‐analysis on the potential efficacy of NIBS for anhedonia symptoms in patients with schizophrenia and depression. Notably, we did not distinguish different kinds of NIBS techniques and stimulus parameters when selecting the studies for meta‐analysis. However, most studies included in our quantitative synthesis involved the use of excitatory stimulations over the prefrontal area, such as high‐frequency rTMS, iTBS, and anodal tDCS. Hence, our results appeared to suggest that enhancing cortical excitability of the prefrontal brain regions could sufficiently improve anhedonia symptoms in patients with schizophrenia and depression. Moreover, other stimulation parameters (such as the number of sessions and stimulation intensity) appeared to influence the effectiveness of NIBS (Bhattacharya et al., 2022). However, we did not examine these potential mediating factors due to the limited number of identified studies.

Rather than being a unitary construct, anhedonia is multi‐dimensional, with at least two components supported by independent neural substrates, namely anticipatory anhedonia and consummatory anhedonia (Su & Si, 2022; Treadway & Zald, 2011). A previous meta‐analysis revealed that anticipatory anhedonia involved dysfunctions in frontal‐striatal networks, whereas consummatory anhedonia involved the ventral basal ganglia area (Zhang et al., 2016). However, all the included studies on schizophrenia patients did not account for possible differential effects and the anticipatory‐consummatory differentiation of anhedonia. Only two included studies on patients with depression investigated the effect of NIBS on anticipatory and consummatory components of anhedonia using the Temporal Experience Pleasure Scale (TEPS) (Duprat et al., 2018; Wang et al., 2021). Wang and colleagues applied rTMS to depression patients with anhedonia symptoms. They found that relative to the sham group, the intervention group significantly improved in anticipatory anhedonia rather than consummatory anhedonia (Wang et al., 2021). However, another study which applied accelerated iTBS (a form of rTMS) found no significant efficacy in either anticipatory or consummatory anhedonia (Duprat et al., 2018). Despite the mixed results, a few studies suggest that rTMS to the dorsolateral prefrontal cortex (DLPFC) could alter dopamine release from the nucleus accumbens, and could modulate reward‐based motivation, which is closely related to anticipatory pleasure (Ahn et al., 2013; Strafella et al., 2001; Wang et al., 2021). In this study, we did not conduct further analysis to verify the efficacy of NIBS on anticipatory and consummatory anhedonia. Future studies could further explore this area.

Despite the popularity of NIBS, medications have remained the first‐line treatment for both schizophrenia patients and patients with depression. It is plausible that NIBS can supplement medications to improve anhedonia, yet very few studies have been conducted to study NIBS–medication interaction. Our systemic review found four studies on schizophrenia patients which did not impose any restriction on medication use (Bodén et al., 2021; Gan et al., 2021; Kumar et al., 2020; Prikryl et al., 2013). Current evidence suggests that atypical antipsychotics are superior to typical antipsychotics in reducing anhedonia symptoms, but neither has reached clinically significant improvement (Fusar‐Poli et al., 2015; Liang et al., 2022). Similarly, for patients with depression, anhedonia is considered to be resistant to antidepressant treatments (Liang et al., 2022), and some types of drugs, such as selective serotonin reuptake inhibitors (SSRIs) or serotonin‐noradrenaline reuptake inhibitors (SNRIs), may even lead to mild anhedonia (Goodwin et al., 2017). In the included RCT studies on patients with depression, all participants went through a 2–6 week period of stable drug use or washout, and this study might control for the effect of the pharmacotherapy (Bodén et al., 2021; Diederichs et al., 2021; Duprat et al., 2018; Jog et al., 2021; Wang et al., 2021; Zhu et al., 2022). However, we did not study the potential impact of drug‐induced anhedonia.

Several limitations should be borne in mind. First, the number of studies included in this review remained small. We did not study confounding factors, such as pharmacological treatment, illness severity at baseline, and different NIBS stimulation parameters. Second, the majority of included studies examined anhedonia as a secondary outcome, and the measurement instruments for anhedonia varied greatly across different studies, including the SHAPS, the TEPS, and items of the BDI and the SANS. Future studies should measure both components of anhedonia as primary outcomes using specific and comprehensive instruments. Third, we did not conduct any pre‐registration for this study. Lastly, we did not include studies which used electroconvulsive therapy (ECT) to treat anhedonia. Having said that, ECT in principle differs substantially from other non‐invasive techniques. ECT uses sedation and induces a grand mal seizure that typically lasts around 20–60 s and affects the whole brain (Trifu et al., 2021); unlike ECT, other NIBS techniques, like TMS and tDCS, target specific areas (e.g. dorsolateral prefrontal cortex, dorsomedial prefrontal cortex) of the brain cortex that are involved with the symptoms. After careful deliberation, we decided not to include ECT in this review and analysis.

Notwithstanding these limitations, we provide preliminary evidence for the beneficial effects of NIBS on anhedonia symptoms in schizophrenia and depression. Given the paucity of previous NIBS studies on anhedonia, our preliminary findings should be interpreted with caution. Given that anhedonia is a transdiagnostic and difficult‐to‐treat symptom, more research using transdiagnostic samples is needed.

## CONFLICT OF INTEREST STATEMENT

The authors declare that there are no conflicts of interest.

## ETHICS STATEMENT

This is a review paper that does not require any ethical issues for approval.

## Supporting information


**Data S1.** Supplementary Information.
