# Supplementary material for: The effect of noninvasive brain stimulation on anhedonia in patients with schizophrenia and depression: A systematic review and meta‐analysis
Source: Psych J. 2023 Dec 27;13(2):166–75. doi: 10.1002/pchj.723 (PMC10990806; doi:10.1002/pchj.723)
Supplement: Supplementary file 1 — Data S1. Supplementary Information. [file PCHJ-13-166-s001.docx]

1. **Search Strategy**

Search performed on: 05-07-2023

**Pubmed**

(anhedonia OR pleasure OR SHAPS OR hedonic) AND (neuromodulation OR "noninvasive brain stimulation" OR "transcranial magnetic stimulation" OR "repetitive transcranial magnetic stimulation" OR "direct transcranial current stimulation" OR “transcranial alternating current stimulation" OR tACS OR tDCS OR TMS OR rTMS) AB/TI

Limits: none

Results: 74

**Web of science**

(anhedonia OR pleasure OR SHAPS OR hedonic) AND (neuromodulation OR "noninvasive brain stimulation" OR "transcranial magnetic stimulation" OR "repetitive transcranial magnetic stimulation" OR "direct transcranial current stimulation" OR “transcranial alternating current stimulation" OR tACS OR tDCS OR TMS OR rTMS) TS

Limits: none

Results: 205

**EBSCOHost (PsycInfo/PsycArticles)**

(anhedonia OR pleasure OR SHAPS OR hedonic) AND (neuromodulation OR "noninvasive brain stimulation" OR "transcranial magnetic stimulation" OR "repetitive transcranial magnetic stimulation" OR "direct transcranial current stimulation" OR “transcranial alternating current stimulation" OR tACS OR tDCS OR TMS OR rTMS) TI,AB

Limits: none

Results: 54

**China National Knowledge Infrastructure**

(快感缺失 + 愉悦情绪 + SHAPS + 愉快感) * (神经调控 + 非侵入性脑刺激 + 经颅磁刺激 + 重复经颅磁刺激 + 经颅直流电刺激 + tDCS + TMS + rTMS + 经颅交流电刺激 + tACS) TI,AB

Limits: none

Results: 27

**Wanfang Data Knowledge Service Platform**

("快感缺失" or "愉悦情绪" or "SHAPS" or "愉快感") and ("神经调控" or "非侵入性脑刺激" or "经颅磁刺激" or "重复经颅磁刺激" or "经颅直流电刺激" or "tDCS" or "TMS"or "rTMS" or “经颅交流电刺激" or "tACS") TI,AB

Limits: none

Results: 15

**Total search results: 74 + 205 + 54 + 27 +15 = 375 articles**

1. Risk of bias assessment

**(A)**

\

**(B)**


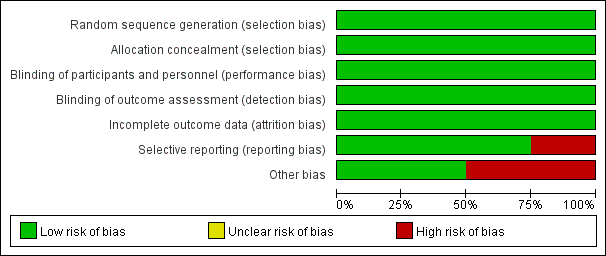


**Figure S1. (A,B) Risk of bias assessment summary on schizophrenia study according to the Cochrane risk of bias tool (A) Risk of bias summary: review authors’ judgments about each risk of bias item for each included study, (B) The risk of bias for the included studies**


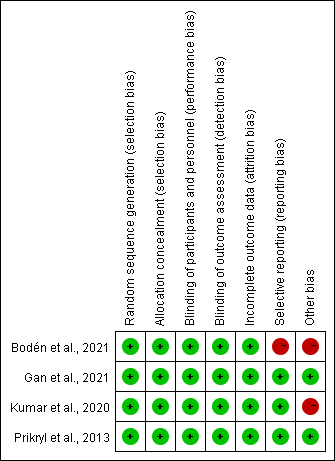


**(A)**


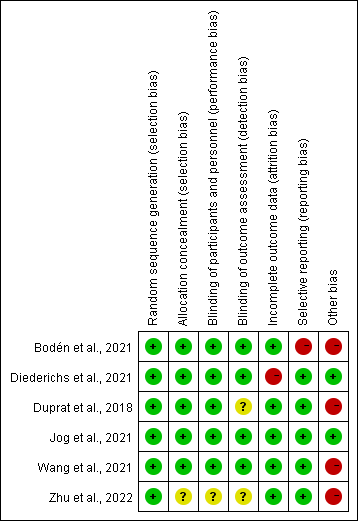


**(B)**


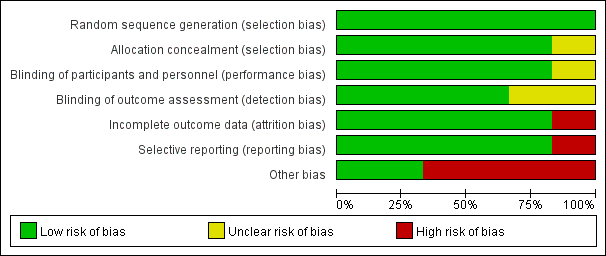


**Figure S2. (A,B) Risk of bias assessment summary on depression study(RCT) according to the Cochrane risk of bias tool (A) Risk of bias summary: review authors’ judgments about each risk of bias item for each included study, (B) The risk of bias for the included studies**

‘

| **Summary of findings** | | | | | **Quality assessment** | | | | | **Quality** |
| --- | --- | --- | --- | --- | --- | --- | --- | --- | --- | --- |
|  | **No. of studies** | **No. of patients** | | **Hedges's**  **(95%CI)** | **risk of bias** | **Inconsistency** | **Indirectness** | **Imprecision** | **publication bias** |  |
|  |  | **Active** | **Sham** |  |  |  |  |  |  |  |
| NIBS for SZ | 4 | 93 | 85 | 0.665  (0.130,1.201) | low^1^ | low^2^ | no Indirectness | low^1^ | low^3^ | very low |
| NIBS for MDD(RCT) | 6 | 144 | 123 | 0.548  (0.315, 0.781) | low^1^ | no Inconsistency | no Indirectness | No Imprecision | low^3^ | low |
| NIBS for MDD(open-label) | 6 | 503 | / | 1.002  (0.656, 1.349) | Low^4^ | low^2^ | no Indirectness | No Imprecision | low^3^ | very low |

Table S1 GRADE assessments

Note: 1. Some of them have risk of bias according to Cochrane risk of bias tool; 2. I^2^>50%; 3. Number of studies is small; 4. the study design(open-label) is was considered to carry some degree of risk of bias
